# HEALTHY AGING: POLICIES AND RESOURCES TO SUPPORT AN AGE-FRIENDLY ECOSYSTEM

**DOI:** 10.1093/geroni/igac059.717

**Published:** 2022-12-20

**Authors:** Patricia D’Antonio, Brian Lindberg

## Abstract

Our public health system is advancing activities to prioritize the health and well-being of older adults and recognizes the importance of policies to support us all as we age. Faculty will discuss national efforts underway to inform and support an age-friendly ecosystem.

